# Murmurs: the journal of art and healing

**Published:** 2019-03-13

**Authors:** Marlon Danilewitz, Andrea Zumrova, Farriss Blaskovits, Lynn Bloom

**Affiliations:** 1 Department of Psychiatry, University of British Columbia, British Columbia, Canada; 2 Faculty of Medicine, University of Ottawa, Ontario, Canada

## Abstract

In an era of unprecedented physician burnout and disconnection, there is growing recognition of the importance of integrating the study of humanities in medicine. Emerging research has underscored the importance of reflective writing and creativity in bolstering physician resilience. The publication “Murmurs Magazine,” therefore, should be of great interest to medical educators. This publication was developed and continues to be managed by medical students as a forum for creative expression and reflection.

## Introduction

Daily practice within clinical settings can be stressful and emotionally demanding, particularly for young medical students at the beginning of their careers. The literary journal as a medium offers medical students a space for the unbridled expression and processing of experiences which can flow freely from the writer’s pen (or computer) directly to the reader. Such creations are not subject to formal grading or assessment and thus are more likely to emerge from the heart. This may be one reason why reflective writing has been shown to benefit student health and wellbeing.^1^^,^^2^ Such a finding is particularly relevant in an era where medical students are experiencing high levels of stress and burnout.^3^ The value of literary journals is not only in the writing, but also in the witnessing of that which is expressed. The less constrained expressions of medical students writing for their peers breaks barriers of isolation, silence, and disconnection.^4^ They see themselves in the writing of fellow students, they take pleasure in the shared experiences that are conveyed, and they acknowledge being part of a broader community of like-minded future physicians and healers. The student journal, “Murmurs,” honors these perspectives by nurturing a forum for open expression and reflection.

### The Publication

“Murmurs” was started in 2012 as a student driven, bilingual literary magazine at the University of Ottawa. While it continues to be a student led project, the publication has continued to expand, attracting submissions from medical trainees, clinicians, and patients from across North America. In 2016 the publication developed a partnership with Shanghai Jiao Tong University School of Medicine leading to the creation of a multilingual publication. In 2018, facilitated by a new French language editor position, the magazine also extended its bilingual reach to numerous Quebec medical schools that had not previously offered submissions.

The magazine publishes a wide range of genres including poetry, fiction, personal narratives, comics, photography, and art. All submissions are peer-reviewed by a team of peer-editors. Acting as peer-editors, they are well poised to select pieces that speak to the medical student experience. Pieces are selected on artistic merit and resonance with the theme selected for each issue. Every year, the magazine has a theme to inspire contributions. A majority consensus is reached by the editorial team prior to including a piece. The editors are often skilled in the visual arts, as well as writing, and bring a passion for creative expression to the crafting of Murmurs Magazine.

### Evaluation of the publication

As a medical profession, there is increasing awareness of the importance of reflection to the empathic delivery of healthcare. “Murmurs” provides an opportunity for its contributors, readers and editors alike to grapple with their moments of greatest vulnerability, strength and human connection. This has led to the growing support of “Murmurs” by both students and educators, as a unique vehicle shaping the experience of medical students in Canada.

Notwithstanding the qualitative significance of the magazine in the eyes of medical students and educators, the publication has a number of limitations. While the magazine publishes submissions in English and French, the majority of contributions are in English and translation is not provided. This makes it more difficult to engage our francophone students. Like other medical student led journals, there is a range in the quality of the contributions varying by year and genre, although the editorial team makes it a point to select the highest quality works possible for each publication. While the focus of the magazine centers on expanding the voice of medical trainees, attracting submissions from allied health professionals would add to the diversity of expression and quality of publication.

The publication possesses a number of strengths, most notably the genuineness of the contributions. The honest content of the contributions provides readers with a window into the lived experience of medical students. Within the pages of “Murmurs,” medical educators will likely find the wide range of themes enlightening, promoting a richer understanding of the dynamic lives of medical trainees. We strongly recommend that medical educators explore this insightful and reflective publication.

## References

[1] Pennebaker J Telling stories: The health benefits of narrative. Literature and Medicine. 2000;19(1):3-18. 10824309 10.1353/lm.2000.0011

[2] Wald HS, Haramati A, Bachner YG, Urkin J Promoting resilience for interprofessional faculty and senior medical students: Outcomes of a workshop using mind-body medicine and interactive reflective writing. Medical Teacher.2016;38(5):525-8. 10.3109/0142159X.2016. 27027210

[3] Jackson ER, Shanafelt TD, Hason O et al Burnout and alcohol abuse/dependence among US medical students. Acad Med. 2016;91(9):1251-6. 26934693 10.1097/ACM.0000000000001138

[4] Hermann N Creativity: What,Why and Where? In: The Principles and Practices of Narrative Medicine. Edited by Charon R, Das Gupta S, Hermann N et al New York: Oxford University Press, 2017.

